# The gene regulatory networks shaping macrophage plasticity and altered function in fibrosis

**DOI:** 10.3389/fimmu.2026.1767954

**Published:** 2026-02-27

**Authors:** Zsuzsanna Kolostyak, Laszlo Nagy

**Affiliations:** 1 Department of Surgery, Transplantation and Gastroenterology, Semmelweis University, Budapest, Hungary; 2 Nuclear Receptor Research Laboratory, Department of Biochemistry and Molecular Biology, Faculty of Medicine, University of Debrecen, Debrecen, Hungary; 3 Departments of Medicine, Pediatrics, Physiology, Pharmacology and Therapeutics and Biomedical Engineering, Johns Hopkins University School of Medicine, Institute for Fundamental Biomedical Research, Johns Hopkins All Children’s Hospital, St. Petersburg, FL, United States

**Keywords:** fibrosis, macrophage dysfunction, macrophage plasticity, tissue microenvironment, tissue remodeling, transcriptional reprogramming

## Abstract

Tissue inflammation and its resolution are fundamental physiological processes that ensure homeostasis and tissue integrity following injury. A precise balance between pro-inflammatory and pro-resolving mechanisms promotes proper tissue repair, whereas dysregulation of these pathways results in chronic damage and fibrosis. This complex multicellular phenomenon ultimately manifests in extensive extracellular matrix (ECM) deposition and organ failure. Although the core transcriptional programs are highly conserved throughout evolution and across different species and tissues, distinct features arise under the influence of specific tissue microenvironments. The functionally divergent phenotypes and widespread heterogeneity of macrophages enable them to play a key modulatory role along the inflammation–resolution–fibrosis axis. Recent advances in epigenetic and transcriptomic profiling have revealed novel regulatory circuits and candidate transcriptional regulators governing macrophage phenotypes in fibrotic contexts. In this review, we aim to integrate current knowledge on the complex, context-dependent regulatory mechanisms and dysfunction of macrophages in fibrosis. We highlight the importance of macrophage ontogeny, signal- and metabolism-dependent transcriptional regulation, and chromatin remodeling in disease progression, with particular attention to therapeutic perspectives.

## 1 Introduction

Fibrosis represents a pathological, maladaptive outcome of the tissue repair program that becomes dysregulated in response to diverse forms of injury, most commonly in the setting of chronic inflammation. Under physiological conditions, the formation of fibrin fibers in association with collagen and fibronectin is a general feature of extracellular matrix (ECM) deposition and constitutes a fundamental step in tissue repair and wound healing across all organs. When tissue damage occurs, macrophages activate local fibroblasts, which initiate the secretion of matrix-assembling proteins. The severity and frequency of tissue insults critically determine the extent and efficiency of the fibrotic process. Transient or limited injuries result in moderate ECM deposition and accumulation, allowing the restoration of normal tissue architecture. In contrast, severe or repetitive insults lead to sustained fibroblast activation, resulting in continuous ECM remodeling and scar formation that progressively disrupt anatomical and functional integrity, ultimately culminating in irreversible organ failure (1).

The trajectory from tissue repair to fibrosis is influenced by numerous factors, including genetic variations, types of exposure, and the activity of the immune system. Among immune cell populations, macrophages play a pivotal role in recognizing external harmful agents, they initiate the inflammatory response and, through dynamic phenotypic changes, mediate the resolution of inflammation and tissue repair. Inadequate regulation of this sensitive phenotypic switch contributes to inflammation-related pathologies such as fibrosis.

The canonical view is that inflammatory macrophages secrete pro-inflammatory cytokines and reactive oxygen species (ROS) to promote pathogen clearance, whereas pro-resolving macrophages orchestrate tissue repair mechanisms (2). This simplified classification underestimates the broad spectrum of macrophage activation states, their remarkable plasticity, and the unique combinations of gene expression patterns underlying these diverse functions. The highly dynamic and signal-sensitive nature of epigenetic regulation allows for fine-tuned control of the inflammatory response. However, maladaptive signaling loops and improper regulatory mechanisms are frequently observed in fibrotic conditions.

Tissue macrophages differ in their ontogeny. They include embryonically derived macrophages originating from yolk sac and fetal liver precursors that give rise to long-lived, self-renewing tissue resident macrophage (TRM) populations, and bone marrow–derived macrophages (BMDMs) that can repopulate tissues from circulating monocytes. All ontogenically diverse populations are involved in fibrotic remodeling, however, the exact effects of these cells largely depend on the local tissue microenvironment (3).

The gene expression and function of macrophages are regulated in three fundamental ways at the molecular level. First, they are shaped by a foundational developmental program that defines cell identity. Beyond this intrinsic layer, transcriptional activity continuously responds to surrounding polarization signals that reflect the local tissue milieu. Finally, the third level of regulation arises upon pathogen exposure or inflammatory stimulation, which triggers a rapid and transient adaptation of gene expression (4).

This regulation is orchestrated through the interaction of lineage-determining transcription factors (LDTFs), including PU.1, C/EBPs, and AP-1 family members (5–7), and signal-dependent factors such as NF-κB, STATs, IRFs, PPARs, and SMADs (2, 7–9). These factors operate within an epigenetic landscape defined by chromatin accessibility, histone modifications, and DNA methylation. Moreover, cellular metabolism adds a complementary regulatory layer, influencing both gene expression and macrophage functionality.

The rapid expansion of multi-omics approaches and single-cell–based transcriptomic and epigenomic technologies, particularly in spatial contexts, has uncovered unprecedented complexity in macrophage heterogeneity and identified novel regulatory circuits underlying their roles in tissue remodeling and fibrosis. Integrated insights from pathologies such as pulmonary, hepatic, renal, and cardiac fibrosis and systemic sclerosis (SSc), provide the foundation for understanding the core and tissue-specific fibrotic programs of macrophages and open new opportunities for the identification of therapeutic targets.

In this review, we summarize how macrophage behavior and contribution to fibrosis is shaped by the integration of developmental origin, injury-derived signals, metabolic regulation, and tissue-specific niche cues. We discuss how ontogeny establishes distinct transcriptional and epigenetic baselines, how damage-associated pathways reprogram macrophage gene expression during acute and chronic injury, and how metabolic state feeds back on chromatin structure and transcriptional control. Finally, we highlight how these regulatory layers influence emerging macrophage-based therapeutic strategies aimed at modifying fibrotic progression and promoting tissue repair.

## 2 The regulatory architecture shaping macrophage identity during fibrosis

### 2.1 Functional macrophage programs across inflammation, resolution and fibrotic remodeling

Tissue macrophages are indispensable regulators of organ integrity, continuously integrating metabolic, mechanical, and stromal cues to maintain homeostasis. Their core effector functions are pathogen and damage recognition through pattern recognition receptors (PRRs), phagocytosis and macropinocytosis, autophagy-driven intracellular quality control, and efferocytosis of apoptotic cells collectively ensure clearance of cellular debris, maintenance of barrier structures, containment of infection, and coordination of stromal–immune interactions (10–12). Through these activities, macrophages orchestrate ECM turnover, modulate vascular integrity, and regulate parenchymal cell survival and metabolism. In a tissue-specific manner, homeostatic macrophage programs support hepatocyte metabolic zonation and bile duct integrity in the liver, regulate surfactant recycling and alveolar epithelial quiescence in the lung, sustain tubular epithelial regeneration and peritubular capillary stability in the kidney, and contribute to cardiomyocyte viability, electrical conduction, and adaptive remodeling in the heart (13, 14).

Macrophage behavior along the inflammation–resolution–fibrosis axis strongly depends on the intensity, repetition, chronicity and tissue context of injury. In acute high-intensity insults such as severe viral pneumonia, toxin-induced liver injury, myocardial infarction or abrupt renal ischemia–reperfusion, macrophages initiate rapid NF-κB-, IRF- and AP-1–driven inflammatory programs that facilitate pathogen elimination, the production of ROS and nitrogen species, the secretion of pro-inflammatory cytokines and chemokines, the recruitment of neutrophils and monocytes, as well as the activation of complement and coagulation pathways, all of which are essential for the early containment of tissue damage (15). These responses are further intensified by inflammasome activation, which drives pyroptotic release of IL-1β and IL-18, and are amplified even more by neutrophil extracellular traps (NETs) (16, 17). NET-derived HMGB1 engages RAGE-dependent pathways in macrophages, induces lysosomal destabilization and cathepsin B release, and activates caspase-1–mediated pyroptosis, thereby further escalating inflammatory cytokine production and sustaining tissue inflammation (16, 17). Functionally, these programs equip macrophages for rapid damage containment and immune amplification, but at the cost of tissue stress and collateral injury.

Recurrent or severe insults can induce a state of trained immunity that preserves chromatin accessibility at inflammatory enhancers and lowers the threshold for reactivation upon subsequent challenges (18). In this heightened inflammatory milieu, pyroptosis- and necroptosis-associated DAMP release further amplifies macrophage activation. In the context of Gram-negative infection and endotoxemia, bacterial lipopolysaccharide (LPS) can access the cytosol of myeloid cells and activate inflammatory caspases. Experimental studies demonstrate that cytosolic LPS sensing induces gasdermin D–dependent pore formation and the extracellular release of the leaderless lectin galectin-1 in macrophages *in vitro*, while systemic LPS challenge triggers a comparable increase in circulating galectin-1 *in vivo*. Genetic deficiency or antibody-mediated neutralization of galectin-1 attenuates cytokine production and improves survival in endotoxemia models, indicating a causal role in amplifying inflammation. Importantly, elevated galectin-1 levels are also detected in the sera of patients with sepsis, supporting the relevance of this pathway in human systemic inflammation (19). These persistent DAMP-driven signals prolong alarmin release, maintain CCL2/CCR2-dependent monocyte recruitment, destabilize metabolic homeostasis and prevent the engagement of resolution programs, ultimately locking macrophages into feed-forward inflammatory circuits that favor profibrotic reprogramming.

Resolution requires efficient efferocytosis, intact autophagic flux and metabolic reprogramming toward oxidative phosphorylation (OXPHOS). Only under these conditions macrophages adopt pro-repair phenotypes that limit ECM deposition and support controlled tissue remodeling (11, 20). In this state, macrophages execute a suite of pro-resolving functions, including the termination of inflammatory cytokine production, the release of IL-10 and specialized pro-resolving mediators, the suppression of inflammasome activity, the normalization of chemokine gradients, the promotion of fibroblast de-activation, and the orchestration of angiogenic regression and matrix remodeling (21). Together, these processes define a functional macrophage state that actively limits fibrotic remodeling and restores tissue architecture.

### 2.2 Comparative framework of fibrotic remodeling across organs

Across organs, macrophages participate in fibrotic remodeling through partially shared biological principles, but the functional outputs of these programs differ depending on tissue architecture, dominant stromal targets and injury dynamics (1, 22). A recurring early feature is chemokine-driven recruitment of circulating monocytes, most prominently via the CCL2/CCR2 axis, which expands macrophage populations capable of sustaining chronic inflammatory and profibrotic signaling (23–25). However, the consequences of this recruitment diverge substantially across organs depending on which stromal cell types serve as the primary fibrotic effectors and how injury signals are spatially and temporally organized (22, 26).

In the heart, TRMs contribute to early injury sensing and monocyte recruitment, but adverse fibrotic remodeling is largely shaped by recruited monocyte-derived macrophages (13, 22, 27). These cells enhance fibroblast activation and ECM deposition through sustained secretion of cytokines such as TGF-β1, IL-1β and TNF, as well as through matrix-remodeling enzymes including MMP-9 (28, 29). Notably, factors classically associated with resolution, such as IL-10, can acquire profibrotic roles in the aging or chronically stressed myocardium, illustrating how conserved signals are reinterpreted by tissue-specific environments (13, 22).

In the liver, macrophage-driven fibrosis is organized around the activation of hepatic stellate cells (HSCs) (30, 31). Kupffer cells act as early amplifiers of injury by releasing inflammatory mediators and chemokines that recruit circulating monocytes (32, 33). During active injury, infiltrating Ly6C^hi^-derived macrophages become the dominant source of inflammatory and profibrotic cues that directly promote HSC activation and collagen deposition (22, 23, 34). At the same time, macrophage programs licensed by IL-4/IL-13–dominated cytokine environment correlate quantitatively with fibrosis severity, yet their functional contribution differs between phases of progression and resolution, underscoring the context dependence of these pathways (35, 36).

Renal fibrosis highlights a distinct macrophage–epithelial–stromal interplay (25, 37). Macrophages accumulate rapidly in response to tubular injury and support fibroblast and pericyte activation through growth factors such as TGF-β1, PDGF and FGF-2 (38, 39). In addition to these paracrine effects, the kidney literature emphasizes epithelial plasticity and the emergence of macrophage-to-myofibroblast transition as organ-relevant mechanisms that reinforce interstitial scarring during chronic disease (40–42). These processes are tightly linked to persistent inflammatory signaling and prolonged macrophage retention within the renal interstitium (43).

In the lung, fibrotic remodeling is strongly influenced by the spatially restricted and repetitive nature of epithelial injury (22, 44, 45). Macrophages accumulate in close proximity to fibroblast foci and sustain profibrotic circuits through mediators such as osteopontin (OPN) and TGF-β/SMAD signaling (46–48). Rather than transitioning through a clearly demarcated inflammatory-to-resolution sequence, lung macrophage populations often display prolonged coexistence of inflammatory, reparative and maladaptive programs, a feature that distinguishes pulmonary fibrosis from more synchronized injury models and contributes to persistent fibroblast activation and matrix deposition (49, 50).

**Figure 1:**
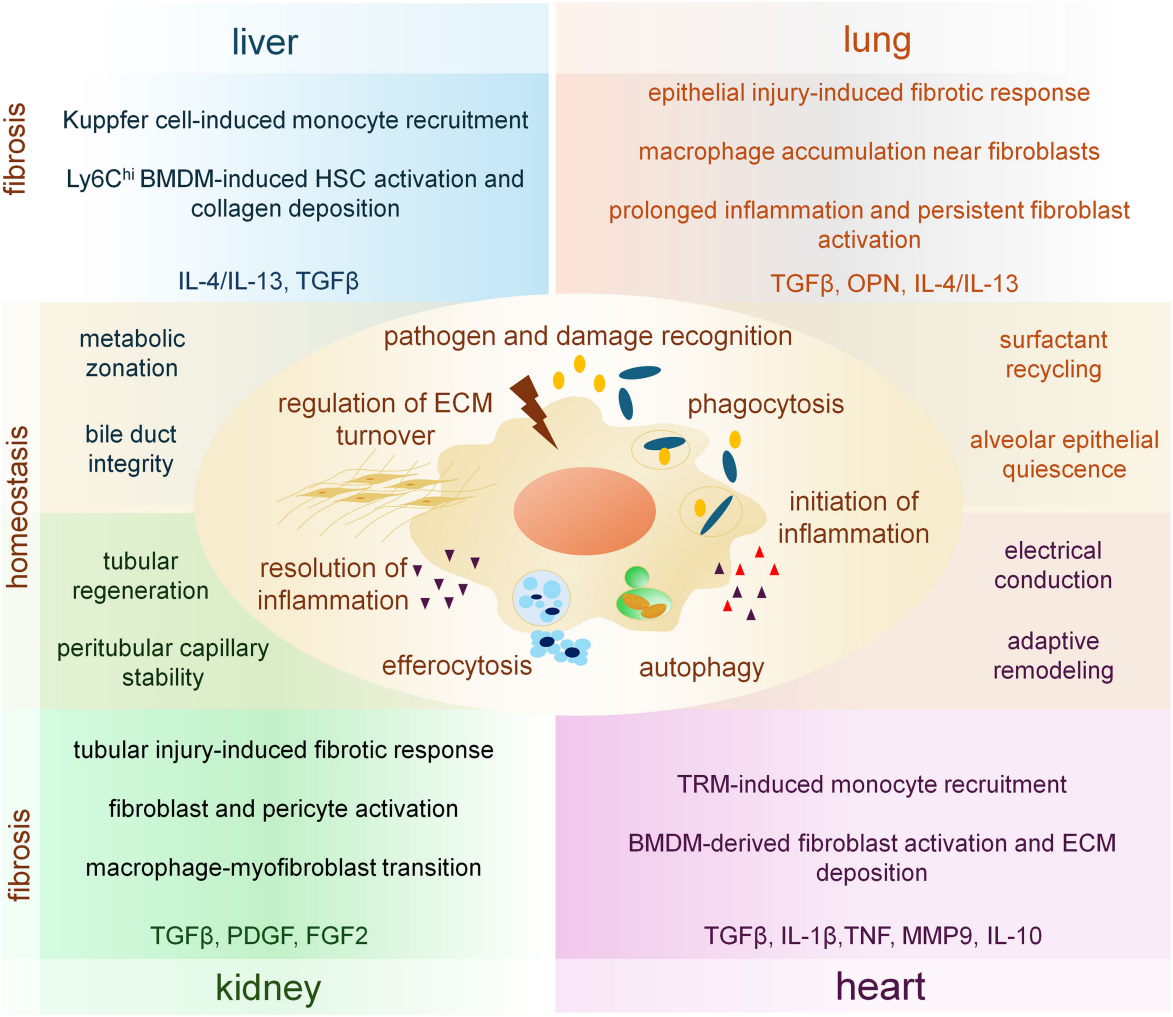
Core macrophage functions and tissue-specific adaptations across fibrotic organs. This schematic illustrates the shared core functions of macrophages and their tissue-specific adaptations in major fibrotic organs. The central panel highlights conserved macrophage activities that operate across tissues. Surrounding panels depict how these core functions are contextually reprogrammed by organ-specific microenvironments during homeostasis and fibrosis (the dominant organ specific fibrotic cytokines are indicated).

Taken together, these observations support a comparative framework in which macrophages do not drive fibrosis through a single universal mechanism. Instead, conserved recruitment and activation principles are redirected by tissue-specific stromal targets, epithelial injury architectures and dominant cytokine milieus. These contextual differences explain why macrophage-driven fibrosis manifests through distinct cellular circuits in the heart, liver, kidney and lung, despite reliance on overlapping molecular inputs. **Figure 1** summarizes the conserved effector functions of macrophages and illustrates how identical functional modules are redirected toward distinct fibrotic outcomes by tissue-specific niches.

However, in many human fibrotic diseases macrophage regulatory programs fail to stabilize in a manner compatible with *ad integrum* tissue repair. In cholestatic liver diseases, such as primary sclerosing cholangitis (PSC), macrophages face repeated cycles of cholangiocyte injury, bile acid toxicity, and microbial products from the gut, which promote pro-fibrotic transcriptional states (51). In metabolic dysfunction–associated liver disease (MASLD) and its inflammatory subtype metabolic dysfunction–associated steatohepatitis (MASH) (52), chronic exposure of hepatic macrophages to lipotoxic intermediates, oxidative stress and apoptotic hepatocytes induces a trained immunity–like state characterized by sustained epigenetic priming and metabolic rewiring. Experimental and human data indicate that oxidized lipids, mitochondrial DAMPs and gut-derived PAMPs promote persistent enhancer accessibility at inflammatory gene loci, coupled to increased glycolytic and mitochondrial activity, thereby lowering the activation threshold for profibrotic cytokine and chemokine production. This maladaptive innate immune memory has been proposed as a key mechanism linking metabolic stress to chronic inflammation and fibrogenesis in MASLD/MASH (18, 32, 53, 54).

Similarly, in the lung, chronic exposure to cigarette smoke, environmental pollutants, or repetitive epithelial injury in diseases such as chronic obstructive pulmonary disease (COPD) and idiopathic pulmonary fibrosis (IPF) disrupts macrophage autophagy, impairs apoptotic cell clearance, and stabilizes macrophage phenotypes that perpetuate fibroblast activation (55). In the kidney, recurrent acute kidney injury (AKI) or chronic metabolic and inflammatory stress drives macrophages toward states characterized by defective resolution and sustained production of pro-fibrotic mediators, contributing to chronic kidney disease (CKD) progression (37). In SSc, macrophages within the skin and internal organs exhibit chronic activation driven by persistent IFN-, IL-6–, and TGF-β–rich environments, promoting stable pro-fibrotic phenotypes that differ markedly from acute wound-healing responses (56).

The specific nature of human injury patterns, whether recurrent subclinical damage (e.g. PSC, CKD), persistent low-grade inflammation (MASLD/MASH, obesity-associated inflammation), or continuous severe injury (IPF, SSc, alcoholic hepatitis) deeply influences macrophage transcriptional states. Importantly, these complex processes, chronic exposures often lack true equivalents in murine models.

In MASLD/MASH, macrophages are exposed to prolonged lipotoxic stress, mitochondrial dysfunction and gut-derived inflammatory cues, which promote durable metabolic and epigenetic reprogramming consistent with trained immunity–like states in both human disease and experimental systems (31, 54). Diet-induced and genetic mouse models reproduce key features of macrophage lipid sensing, inflammasome activation and cytokine production, yet differences in lipid species, disease duration and systemic metabolism limit their ability to fully capture the long-term macrophage imprinting observed in human MASLD/MASH (34, 57).

In contrast, cholestatic liver diseases such as PSC are characterized by repeated, spatially restricted cholangiocyte injury, bile acid–mediated toxicity and chronic immune activation, which collectively drive sustained macrophage recruitment and stabilization of profibrotic transcriptional programs over many years (51, 58). Notably, no experimental model fully recapitulates the human PSC disease course. While the Mdr2-/- mouse reproduces aspects of biliary injury and periductal fibrosis, it lacks the immune-mediated and episodic injury patterns that define human PSC, limiting its utility for modeling macrophage dysfunction in this setting (51, 59).

A similar disconnect between human disease and experimental injury paradigms is evident in other organs. In IPF, persistent, spatially confined alveolar epithelial injury promotes long-term retention of monocyte-derived macrophages with stable profibrotic identities that accumulate in fibroblast-rich niches (48, 49, 55). By contrast, bleomycin-induced lung injury captures early inflammatory macrophage activation but fails to reproduce the prolonged coexistence of inflammatory, reparative and maladaptive macrophage programs characteristic of human IPF (60). Likewise, in CKD, recurrent injury and metabolic stress stabilize macrophage states that resist resolution and promote interstitial fibrosis, whereas commonly used unilateral ureteral obstruction (UUO) models reflect acute, synchronized injury with limited relevance to the chronic human condition (42, 43).

Trained immunity adds another layer of complexity, particularly relevant in chronic human diseases. Long-term exposure to metabolic stress, dietary lipids, pollutants, or recurrent sterile inflammation leads to durable epigenetic and metabolic reprogramming in macrophages and their progenitors (18). While such changes may be adaptive in protecting against repeated microbial threats, in MASLD/MASH, COPD, CKD, and SSc-like environments this persistent reprogramming biases macrophages toward exaggerated inflammatory or pro-fibrotic trajectories, thereby accelerating tissue remodeling. Importantly, trained immunity is markedly more pronounced in humans due to longer lifespan, cumulative environmental exposures, and complex comorbidities, factors that mouse models can only approximate.

Macrophage function across the inflammation–resolution–fibrosis continuum emerges from the interplay of their diverse effector mechanisms, metabolic and epigenetic adaptability, and the complex nature of human tissue injury. These features allow macrophages to manage acute damage effectively but expose a vulnerability during chronic or repetitive injury, where regulatory checkpoints fail to reset and macrophages become fixed in pathogenic states. Understanding how these functional programs are engaged, sustained or fail is essential for dissecting the multilayered regulatory systems that govern macrophage behavior in fibrosis.

### 2.3 Developmentally encoded transcriptional programs establishing macrophage identity

TRMs acquire their identity through a transcriptional hierarchy that is installed during embryonic development and subsequently reinforced by tissue-derived cytokines and metabolic cues, creating a stable and highly specialized epigenetic landscape that persists throughout life. At the top of this hierarchy stands PU.1, the essential pioneer transcription factor that binds nucleosomal DNA in yolk sac–derived erythro-myeloid progenitors and fetal liver monocytes, initiating macrophage-lineage specification by opening previously inaccessible chromatin (61–63). PU.1 recruits IRF8, C/EBPα/β, RUNX1 and members of the MITF/TFE family to thousands of enhancer candidates, consistent with enhancer-mapping and cooperative TF-binding analyses (64, 65). This early cooperative pattern draws in chromatin remodelers such as BRG1 and histone acetyltransferases including p300/CBP, generating the H3K4me1- and H3K27ac-marked enhancer scaffold (4).

As macrophages settle into developing organs, PU.1-established enhancer scaffolds are shaped further by cytokines and growth factors emanating from the tissue niche, triggering a second wave of transcription factor recruitment that defines tissue-resident identity. CSF1R signaling, driven by CSF1 or IL-34 from stromal and parenchymal cells, activates a MAPK–AP-1 axis and induces MAF and MAFB, which stabilize macrophage differentiation and repress inappropriate inflammatory circuits (66–68). Parallel to CSF1, TGF-β emerges as a dominant imprinting cytokine in many tissues. SMAD2/3 binding to PU.1-primed enhancers recruits KMT2D and p300, promotes H3K27ac deposition, and locks in tissue-specific gene-expression modules (65, 69, 70). Enhancers established under CSF1, TGF-β and AP-1 remain accessible and actively maintained throughout adult life, reflecting the “epigenetic permanence” of TRM identity (4, 64, 65).

TRMs acquire enhancer landscape and 3D chromatin architecture within a highly permissive epigenetic environment that supports large-scale enhancer establishment, nucleosome repositioning, and topologically associated domain reorganization. These niche-derived cues generate highly specialized enhancer landscapes that remain stable throughout life and profoundly shape how each TRM population interprets inflammatory and fibrotic signals.

BMDMs lack the entire embryonic imprinting process. Their identity is driven largely by PU.1 and C/EBPβ under M-CSF influence, producing a macrophage-like program without the dense enhancer architecture of TRMs (64, 65, 71). Accordingly, although PU.1 is common to both TRMs and BMDMs, in TRMs it cooperates during embryogenesis with other pioneer transcription factors such as IRF8, RUNX1 and MITF/TFE to build stable, tissue-imprinted enhancer landscapes, whereas in BMDMs PU.1 mainly supports macrophage identity and relies on inducible partners such as NF-κB, AP-1, STATs and SMADs to drive context-dependent reprogramming (63, 64, 72, 73). BMDMs retain a far more malleable chromatin landscape, relying heavily on external stimuli (e.g. LPS, IL-4, IFN-γ, TGF-β) to remodel enhancers through inducible p300 recruitment and H3K27ac gain (72, 73). Even under strong cytokine stimulation, BMDMs only partially approximate TRM identity because they cannot recreate the pioneer-factor–dependent enhancer establishment that occurs uniquely during embryogenesis. Although adult BMDMs generally display more constrained chromatin remodeling upon tissue entry, under conditions in which the niche becomes available they can undergo substantial transcriptional and epigenetic reprogramming and differentiate into fully functional, self-renewing TRMs, as demonstrated for Kupffer cells (74).

Several studies report that BMDMs recruited to injured tissues often acquire pro-fibrotic gene expression profiles and exhibit considerable chromatin and transcriptomic plasticity, suggesting that BMDMs may have a greater propensity to adopt pro-fibrotic transcriptional states (75–77). Consequently, mechanistic insights derived from BMDMs cannot be directly extrapolated to TRMs, and developmental embedding must be considered when interpreting macrophage reprogramming in fibrosis or designing macrophage-targeted therapies.

### 2.4 Chromatin landscape, cytokine signaling and tissue context as determinants of macrophage plasticity and fibrotic fate

Macrophage responses to tissue injury are orchestrated by a conserved transcriptional architecture that integrates signals from PRRs, cytokine pathways, metabolic stress, and the tissue niche. The earliest phase of injury sensing begins when TLRs, CLRs or NLRs detect pathogen- or damage-associated ligands, activating NF-κB, AP-1 and IRF transcription factors. These pathways induce rapid inflammatory programs. The ability of these transcription factors to exert broad regulatory effects stems from their capacity to access PU.1-primed enhancers, which are enriched for H3K4me1 and readily accommodate stimulus-dependent chromatin opening. Sustained, non-oscillatory NF-κB activation can disrupt nucleosomal barriers and convert latent enhancers into fully active regulatory sites, enabling robust induction of *Il1b*, *Tnf*, *Cxcl1* and other early-response genes (78, 79). Macrophage activation across diverse tissues and inflammatory contexts nonetheless follows a limited number of conserved transcriptional trajectories, particularly in infiltrating monocyte-derived populations. These trajectories converge on four recurrent activation paths. Phagocytic, inflammatory, oxidative stress–responsive, and remodeling—providing ones are a unified framework for interpreting signal-dependent macrophage states (79). Importantly, the ability of external cues to reprogram macrophage chromatin is not uniform. The ability of NF-κB to activate latent enhancers depends on the temporal dynamics of its signaling, with sustained, non-oscillatory activation uniquely capable of disrupting nucleosomal barriers and converting latent enhancers into fully active regulatory sites (80). Together, these early signaling events define a conserved transcriptional entry point through which macrophages interpret diverse forms of tissue injury. In parallel, MAPK pathways stimulate AP-1 family dimers, which cooperate with NF-κB at numerous inducible enhancers to remodel chromatin, recruit p300/CBP and BRG1-containing SWI/SNF complexes and generate *de novo* H3K27ac deposition essential for early inflammatory gene induction (81). TRIF-dependent phosphorylation of IRF3 and IRF7 provides an additional axis of injury sensing that integrates necrosis, viral, and cytosolic DNA signals into the transcriptional response, forming the canonical type I interferon module (82). These early transcription factors constitute a universal “first wave” of transcriptional remodeling. As the injury response progresses, macrophages integrate cytokine-derived signals that further shape their transcriptional trajectories. IFN-γ-activated STAT1 amplifies inflammatory gene expression by enhancing accessibility at IRF1, NF-κB and AP-1–occupied enhancers (83, 84).

Conversely, IL-10–induced STAT3 acts as a central anti-inflammatory switch, displacing co-activators and promoting repressive chromatin environments that limit further inflammatory activation (85–87). IL-4/IL-13–mediated STAT6 activation represents another conserved pathway, capable of establishing enhancer accessibility associated with repair and metabolic reprogramming (88). IL-4–activated STAT6 binds previously inaccessible genomic regions to establish *de novo* enhancers that support tissue-repair and metabolic programs, while simultaneously repressing inflammatory loci through reduced p300 occupancy and HDAC3 engagement (89).

EGR2, induced downstream of IL-4–STAT6, acts as a late integrator of repair-associated cues (90). Insufficient EGR2 activation fails to imprint a reparative transcriptional program, promoting chronic inflammation and tissue scarring in multiple injury models (91–94). Both IL-4 and glucocorticoids are capable of inducing pro-resolving macrophage states, even though they rely on entirely different upstream signaling mechanisms. IL-4 initiates a STAT6-dependent remodeling program that gradually establishes sustained and late enhancers enriched for EGR2 binding and creates a stable reparative transcriptional network. Glucocorticoids act through ligand-activated glucocorticoid receptor (GR), which binds directly to genomic response elements and recruits GRIP1, a cofactor that promotes broad chromatin opening and activates many of the same regulatory regions that appear during IL-4 polarization. Despite the mechanistic differences, experiments in BMDMs show extensive overlap between IL-4-induced and glucocorticoid-induced transcriptional programs, including the activation of genes involved in phagocytosis, inflammatory restraint and tissue repair (95). These observations indicate that glucocorticoids, similar to IL-4, impose a durable epigenomic configuration that supports resolution-oriented macrophage behavior rather than inflammatory persistence.

TGF-β–activated SMAD2/3 complexes cooperate with AP-1 and PU.1 at context-specific enhancers, shifting gene expression toward matrix remodeling and fibroblast–macrophage communication (96). Recently found that myeloid-specific deletion of TGFBRI exacerbates diet-induced MASH, leading to more severe hepatocyte injury, inflammation and fibrosis. TGFBRI-deficient macrophages also exhibit transcriptional signatures consistent with enhanced inflammasome activation, heightened cytokine signaling, increased cellular senescence, and a more immunosuppressive profile (97).

The termination of inflammatory programs relies on factors such as ATF3 and NR4A1, which recruit HDAC complexes and promote enhancer decommissioning (98, 99). When these resolution checkpoints fail, macrophages retain an aberrant enhancer landscape that sustains low-level inflammatory activity and predisposes to fibrosis. In LPS-induced sepsis model, ATF3 deficiency resulted in enhanced alveolar macrophage pyroptosis (100). ATF3 also regulates interferon dynamics by restraining both basal and inducible IFN-β production through direct binding to a distal regulatory element of the *Ifnb1* locus. Because ATF3 is itself induced by type I interferons, it forms a negative feedback loop that dampens excessive IFN-driven transcriptional programs. Loss of this regulatory circuit enhances IFN-β output and amplifies downstream interferon-responsive genes, creating an inflammatory milieu that favors chronic immune activation and increases the risk of fibrosis under repeated or unresolved tissue injury (101).

Beyond the early cytokine-driven programs, macrophages also engage conserved stress- and metabolic-sensing transcription factors that help them interpret hypoxia, ER stress, and lipid availability during injury. Although the detailed mechanics of these modules differ across tissues, factors such as HIF1α, ATF4, XBP1 and lipid-sensing nuclear receptors (PPARγ/PPARδ/LXRα) form a shared regulatory layer that integrates metabolic cues with inflammatory thresholds (102–104).

The impact of these conserved mechanisms becomes fully apparent only when viewed within the context of tissue-specific niche imprinting. Each organ imparts a unique constellation of transcriptional cues that preconfigure enhancer accessibility long before injury occurs. These niche-derived programs determine which inducible enhancers exist, how accessible they are to inflammatory TFs, and whether injury signals are translated into repair or fibrosis.

In the lung, GM-CSF and surfactant lipids shape a PPARγ- and C/EBP-centered enhancer network that maintains oxidative homeostasis and limits inflammatory amplitude (105–107). The liver niche, enriched in bile acid metabolites, microbial ligands and lipid-rich hepatocyte products, generates an LXRα- and FXR-dominated enhancer framework that promotes detoxification and immune tolerance. Renal macrophages, preconditioned by tubular epithelial signals, complement fragments and high osmolarity, rely on IRF4-, and PPARδ-dependent enhancers that support epithelial communication and immunoregulation (108–110). Under sustained hypoxia or metabolic stress HIF1α and ATF4 become dominant drivers (111). Similarly, cardiac macrophages, shaped by biomechanical forces and cardiomyocyte-derived CSF1, maintain a homeostatic enhancer architecture built around GATA6, MEF2C and NR4A receptors (112–114). Chronic mechanical strain or microvascular ischemia disrupts this architecture and biases macrophages toward SMAD3- and AP-1-driven remodeling programs that support fibroblast activation. Comparable principles apply to dermal and skeletal muscle macrophages, where chronic IL-33, TGF-β, hypoxia or mechanical stress convert tolerance-biased enhancer networks into fibrosis-permissive landscapes (11).

**Figure 2:**
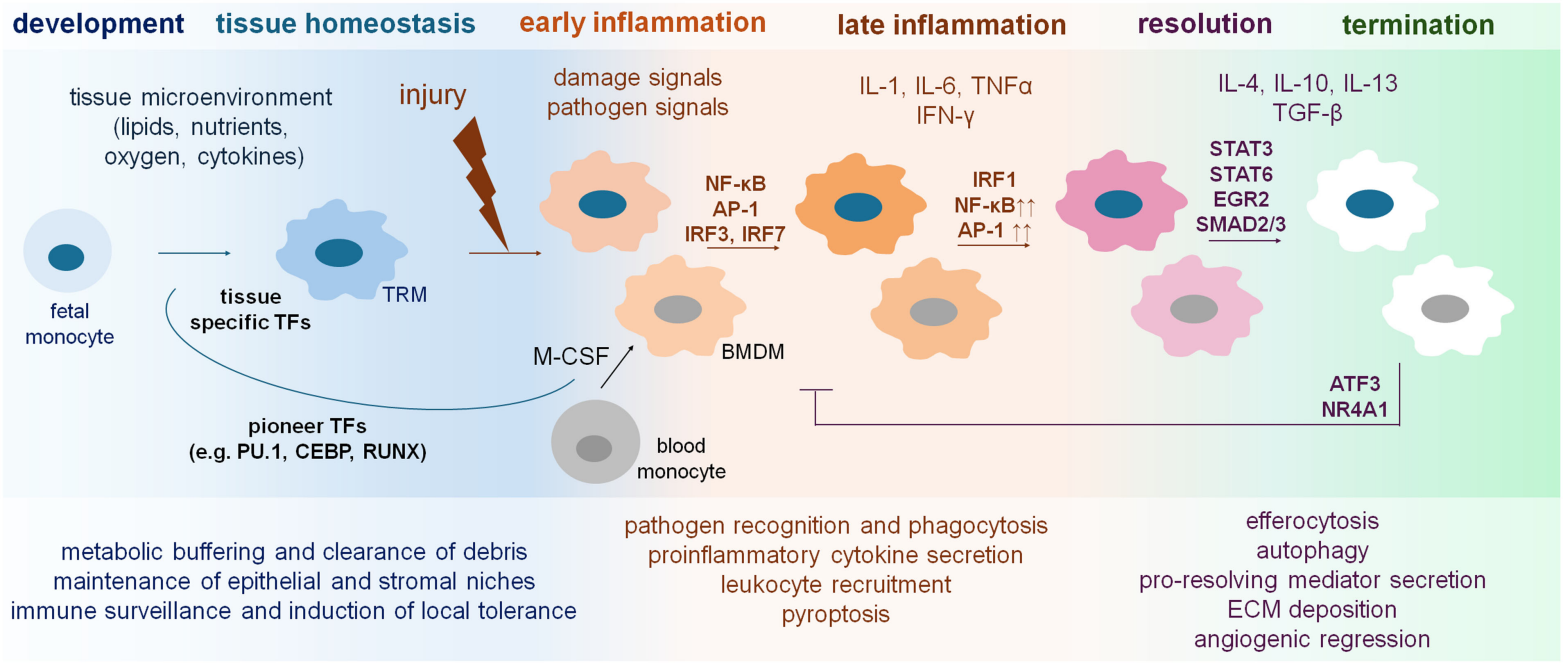
Key signals and transcriptional programs shaping macrophage functions in homeostasis, inflammation and resolution. The figure summarizes major environmental inputs and transcription factors that govern macrophage behavior across activation states. In homeostasis, macrophages support tissue integrity through clearance and metabolic regulation. Upon injury, PRR-derived signals activate NF-κB, AP-1 and IRFs to drive inflammatory responses. Successful resolution engages specialized pro-resolving mediators and repair-associated programs that restore tissue homeostasis.

Ultimately, fibrosis arises when the balance between conserved signal-dependent transcriptional programs and the tissue-specific mechanisms that normally enforce resolution become destabilized. NF-κB, AP-1, IRFs and STAT1 provide essential host-defense functions, yet in the absence of timely STAT3, STAT6, ATF3 and NR4A1 activation, these same pathways drive macrophages into persistent inflammatory–remodeling states. Tissue niche cues dictate which enhancers are available, metabolic and mechanical stresses shape chromatin responsiveness, and recurrent injury imprints long-lasting transcriptional memory. When these layers converge under maladaptive conditions, macrophages lose the capacity to return to a reparative state and instead become central drivers of fibroblast activation and ECM accumulation across organs. These regulatory principles, integrating ontogeny, environmental signals, transcriptional control and functional outcomes across homeostasis, inflammation and resolution, are summarized in **Figure 2**.

### 2.5 Metabolism as a structural determinant of macrophage transcriptional states

Macrophage transcriptional plasticity is tightly integrated with the metabolic reprogramming that accompanies injury, inflammation, and tissue stress. Far from being passive supporters of bioenergetic needs, metabolic pathways actively shape transcriptional outputs through substrate-level control of chromatin modifications, modulation of transcription factor activity, and cofactor redistribution. One of the earliest mechanistic demonstrations of this concept came from the seminal work of Tannahill et al., showing that succinate accumulation during LPS stimulation stabilizes HIF1α and directly drives *Il1b* transcription (102). This finding established that metabolic intermediates could function as *bona fide* signaling molecules in macrophage activation.

Glycolytic reprogramming constitutes a core part of this metabolic–transcriptional circuitry (115). Activation through LPS/IFN-γ or TLR2/3/4/9 produces nearly identical metabolic flux patterns, whereas IL-4–driven activation induces only minor metabolic changes. The enhanced glycolysis observed during classical activation is supported by a switch from the liver-type PFK2 isoform to the more active ubiquitous PFK2, a process partly dependent on HIF-1α but maintained through additional HIF-1α–independent mechanisms (116). In parallel with these early glycolytic adaptations, macrophages rapidly divert glucose-derived carbons into acetyl-CoA to support chromatin acetylation during the first hours of TLR4 stimulation. This metabolic rerouting increases *de novo* H3 acetylation, particularly at H3K27, and prepares inducible enhancers for transcriptional activation even before late inflammatory pathways emerge.

As inflammation progresses, citrate metabolism bifurcates toward distinct transcriptional outcomes. Acetyl-CoA generation promotes early gene induction, whereas subsequent conversion of citrate into itaconate acts as a delayed negative-feedback signal that suppresses multiple cytokines. ACLY activity is indispensable for these early chromatin changes, and its inhibition selectively impairs late-phase inflammatory genes such as *Il6, Il12b, Il18* and *Cxcl9/10* while sparing rapid-response loci (117). Recent work further expands this model by showing that glycolytic enzymes themselves can shape the anti-inflammatory output of macrophages. Pharmacologic activation of PKM2 into its tetrameric form enhances IL-10 production during LPS stimulation by diverting glycolytic flux toward ATP release, which is subsequently converted to adenosine and signals through A2a receptors to reinforce IL-10 expression. These observations indicate that glycolysis does not exclusively support inflammatory activation but can also generate metabolites that actively drive anti-inflammatory and pro-resolving cytokine production (118).

Mitochondrial metabolism exerts similarly important transcriptional effects. IL-4 signaling activates the Akt–mTORC1 pathway in parallel with the canonical JAK–STAT6 axis, enabling metabolic inputs to shape a defined subset of IL-4–induced transcriptional programs. Through coordinated control of ACYL expression and phosphorylation, this pathway increases acetyl-CoA availability and supports p300-dependent histone acetylation at selected enhancers. Physiological mTORC1 activity facilitates this coupling, while chronically elevated mTORC1 impairs IL-4–driven polarization, mirroring metabolic stress responses in other tissues (119). In contrast, α-ketoglutarate (α-KG) generated through glutaminolysis or intact TCA flux, acts as a cofactor for TET DNA demethylases and JmjC-domain histone demethylases such as KDM6A/UTX. The importance of α-KG in promoting resolving transcriptional states was highlighted by Liu et al., who showed that increased α-KG supports H3K27me3 demethylation at STAT6-responsive enhancers following IL-4 stimulation (120). These observations established that the ratio of succinate to α-KG serves as a metabolic “toggle” that biases macrophages toward inflammatory versus reparative gene-expression programs. Together, these studies established that central carbon metabolism functions as a bidirectional switch that tunes macrophage inflammatory versus resolving transcriptional programs.

Fluctuations in lipid availability and composition act as upstream determinants of macrophage enhancer activation and transcriptional fate as well. LXRs, activated by oxysterols, promote cholesterol efflux through genes such as *Abca1* and *Abcg1* (121). They also restrain inflammatory gene expression by maintaining NCoR and SMRT co-repressor complexes at NF-κB–responsive loci, thereby limiting enhancer activation during TLR-induced signaling (63). PPARγ and PPARδ respond to fatty acid–derived ligands and further stabilize anti-inflammatory transcriptional programs by recruiting p300 to oxidative and lipid-handling genes while preventing co-repressor removal at selected inflammatory enhancers (107, 122). Beyond these ligand-driven functions, PPARγ also exerts a distinct ligand-insensitive role during IL-4–induced activation. In this setting, PPARγ binds DNA in the absence of exogenous ligands and recruits p300 together with the architectural protein RAD21, which creates a permissive chromatin state that enables efficient re-engagement of STAT6 and RNA polymerase II during secondary IL-4 exposure. This mechanism establishes transcriptional memory and strengthens the induction of ECM–remodeling genes that are activated during tissue repair, including in IL-4–rich environments such as regenerating skeletal muscle (123). GR signaling adds an additional layer of control by blocking NF-κB access to co-activators and by guiding co-repressors such as NCoR back to AP-1– and NF-κB–occupied sites, producing a strong repression of pro-inflammatory transcription (124).

Amino acid metabolism modulates transcriptional states through several conserved mechanisms. The classical iNOS–arginase axis provides a direct link between arginine flux and transcriptional polarization by influencing the DNA-binding of IRF5 (125). Methionine metabolism regulates the availability of S-adenosylmethionine, the universal methyl donor for DNA and histone methylation. Altered S-adenosylmethionine availability shifts DNA and H3K4 methylation dynamics at inducible enhancers, shaping inflammatory responsiveness (126, 127).

Together, these experimental observations define that macrophage metabolism is not downstream of activation but a primary structural determinant of transcriptional state. Metabolism acts directly on chromatin modifiers, transcription factor binding, and enhancer architecture, enabling macrophages to rapidly transition among states in response to tissue injury.

### 2.6 Metabolic tissue niches and macrophage adaptation in fibrosis

The unique metabolic conditions present within each organ, including oxygen tension, lipid composition, mitochondrial stability, osmotic pressure and nutrient availability, continuously shape enhancer architecture. These influences operate across all macrophage lineages and manifest in the mechanisms of fibrotic diseases.

The lung provides a uniquely high oxygen and low glucose microenvironment that imposes distinct metabolic constraints on alveolar macrophages. In the alveolar lumen the oxygen tension approaches 100 mmHg while glucose levels remain almost ten times lower than in the blood, which forces TRMs to rely on specialized nutrient handling and substantial fatty acid oxidation (FAO) supported by surfactant-derived lipids (128). Under homeostasis these cells depend on glycolysis to maintain immune quiescence and to restrain type 2 inflammation, and allergen exposure further increases glycolytic flux in a TLR2 dependent manner, illustrating the dynamic metabolic plasticity of alveolar macrophages (129). In fibrotic lung disease including IPF, macrophages undergo extensive metabolic remodeling with enhanced glucose uptake, elevated expression of GLUT1, and intensified glycolytic activity, all of which fuel NADPH production and promote NOX mediated ROS formation (130).

Mitochondrial dysfunction is a consistent feature of this state, with IPF macrophages showing dysmorphic mitochondria, increased mitochondrial ROS, reduced expression of OXPHOS genes, and impaired mitophagy due to loss of PINK1, PARK2 and NRF1 (131). Levels of the endogenous anti-fibrotic metabolite itaconate are also reduced because ACOD1 expression is diminished, and this enhances susceptibility to pro-fibrotic activation, whereas exogenous itaconate restrains fibroblast activity (132).

Lipid handling is also profoundly disrupted because epithelial injury releases excess surfactant lipids and oxidized phospholipids into the alveolar space, which leads to foam cell formation and increased production of TGF-β; impaired lipid efflux due to loss of ABCG1 worsens fibrosis, while GM-CSF improves lipid clearance and reduces pathology (133). Additional layers of metabolic dysregulation arise through activation of PERK, which enhance FAO in macrophages and promote fibrotic remodeling (134). Single-cell and lipidomic studies also identify TREM2^+^ monocyte-derived macrophages as important lipid sensing and chemokine producing populations with altered sphingolipid metabolism, and experimental targeting of TREM2 mitigates fibrosis (135). These observations together show that the distinctive metabolic landscape of the lung shapes macrophage behavior and that the progression of fibrosis reflects converging disturbances in glycolysis, mitochondrial homeostasis, redox balance, lipid metabolism and sphingolipid signaling.

The liver’s strong metabolic zonation creates characteristic oxygen, nutrient and lipid gradients, and these cues shape how hepatic macrophages adapt during steatohepatitis and fibrosis (136). During injury, TREM2^+^ BMDMs accumulate around damaged hepatocytes where they help process excess lipids and support ECM remodeling. The loss of TREM2 disrupts lipid handling and results in more severe inflammation, cell death and fibrosis. Circulating soluble TREM2 also reflects disease severity in human MASH (137). As steatohepatitis advances, hepatic macrophages increasingly rely on glycolysis, a shift driven by PKM2 mediated activation of HIF1α, and this metabolic program promotes inflammatory polarization and fibrogenesis, while experimental inhibition of PKM2 nuclear functions alleviates disease in mice (138–141).

Lipid overload further shapes macrophage identity by driving the emergence of lipid-associated macrophages (LAMs) that initially buffer lipotoxic stress but, as efferocytosis and β-oxidation decline, acquire a more inflammatory and pro-fibrotic phenotype (142–145). Additional metabolic stress pathways modify macrophage behavior, since activation of the IRE1α–XBP1 branch of the ER-stress response reinforces inflammatory programming and NLRP3 activation, and impaired canonical autophagy enhances macrophage-driven fibrogenesis (146–150).

Iron accumulation is also common in chronic liver disease and promotes oxidative stress and pro-fibrotic macrophage states. Nuclear receptors act as important metabolic integrators in this environment. PPARδ and PPARγ enhance cholesterol efflux, OXPHOS and glutamine use, thereby supporting anti-inflammatory and restorative macrophage functions, while LXR activation increases lipid efflux and suppresses glycolysis, limiting inflammatory activation (121, 151–158). FXR signaling also contributes to anti-inflammatory polarization, and myeloid FXR deficiency worsens liver injury and diminishes therapeutic responses to FXR agonists (58).

Renal fibrosis develops in a setting where injured tubules, altered metabolites, and hypoxic tissue niches continually influence how macrophages respond to damage. Single-cell analyses suggest that macrophages in fibrotic kidneys shift toward a more glycolysis-dependent state, and experimental inhibition of the glycolytic regulator PFKFB3 limits their accumulation and reduces the inflammatory and matrix-remodeling programs that sustain fibrosis. These findings indicate that glycolytic metabolites help stabilize HIF1α and push macrophages into states that promote scarring (159).

The injured kidney also shapes macrophage behavior through its own metabolic signals. One example is transglutaminase TG2, whose increased activity encourages macrophages to adopt a repair-oriented profile in response to IL-4, yet during chronic injury these cells switch roles and begin to drive fibrotic tissue buildup, TG2 inhibition reduces their numbers and improves kidney structure (160).

Mitochondrial balance is equally important. Loss of the mitochondrial regulator NLRX1 causes macrophages to adopt a highly oxidative and TGFβ-producing state that worsens tubular injury and accelerates fibrosis (161). Tubular cells further influence macrophages through metabolites they release. Elevated levels of the gut-derived compound trimethylamine-N-oxide (TMAO) increase lactate production in tubular cells, and this lactate modifies macrophage chromatin through H4K12 lactylation in a p300-dependent manner, enhancing glycolytic and pro-fibrotic gene expression. Blocking TMAO protects against renal fibrosis and parallels clinical observations (162). Lipid metabolism contributes as well. Loss of the lipid-processing enzyme ACOT12 leads to lipid accumulation, impaired peroxisomal turnover and more severe fibrosis (163).

The healthy heart relies mainly on FAO, yet under pressure overload or myocardial infarction it shifts toward greater glucose use. Cardiac fibroblasts follow the same trajectory and become increasingly glycolytic and glutamine dependent, a change that supports their transition into collagen-producing myofibroblasts (112, 164, 165). In experimental infarction, these glycolytic programs become highly pronounced. Chen and colleagues showed that when fibroblasts from infarcted hearts were exposed to the glycolysis inhibitor 2-deoxyglucose, their glycolytic enzymes and fibrotic markers declined and collagen deposition was reduced, although high systemic doses caused early post-infarct mortality, emphasizing the importance of targeted delivery (166).

Macrophages undergo a parallel metabolic shift. Injured myocardium drives them toward enhanced glycolysis and pentose phosphate pathway activity, increasing ROS and cytokine production and amplifying fibroblast activation (164, 167). A second key experiment demonstrated that when 2-deoxyglucose was delivered locally through a chitosan-based patch applied to the infarct area, macrophages showed reduced NF-κB and NLRP3 activity, the inflammatory milieu softened, and cardiac function improved (168).

Other studies reveal how tuning macrophage metabolism can influence healing. Inhibition of GSK3β increases glycogen turnover and activates TFEB, which enhances glucose uptake in macrophages and shifts them toward a state that restrains fibroblast activation and limits fibrosis (166, 169). Hypoxic macrophages can also mitigate fibrosis by inducing oncostatin M through HIF1α, which blunts TGF-β1 signaling in fibroblasts (52). The epigenetic regulator NPM1 acts in the opposite direction. It sustains mTOR-driven inflammatory glycolysis in macrophages, and its inhibition redirects them toward mitochondrial ATP production, reduces scar formation, and improves ventricular remodeling after infarction (75).

In SSc, dermal fibroblasts adopt a distinctly pro-fibrotic metabolic state. TGF-β1 increases glycolysis and succinate production and blocking glycolysis with 2-deoxyglucose or 3PO markedly reduces collagen expression. Fibroblasts from patient lesions also depend more strongly on glutaminolysis and glutaminase inhibition dampens TGF-β1-driven fibrotic signaling. Itaconate has the opposite effect of succinate and lowers collagen levels in SSc fibroblasts (1). Insights from autoimmune macrophage biology show how metabolic priming toward glycolysis and reduced oxidative metabolism can sustain chronic inflammation, a pattern that may reinforce the pro-fibrotic microenvironment in SSc (170). These metabolic programs that shape macrophage transcriptional states and fibrotic outcomes are summarized in **Table 1**.

**Table 1:** Metabolic signals linking chromatin regulation to macrophage states in fibrosis.

Metabolic signal	Epigenetic coupling	Macrophage state outcome	Fibrotic context
succinate accumulation	HIF1α stabilization and *Il1b* enhancer activation	acute inflammatory macrophage activation	lung, liver, kidney
glucose-derived acetyl-CoA	ACLY-dependent H3K27 acetylation at inducible enhancers	primed but reversible inflammatory state	general
itaconate production	NRF2 activation and suppression of cytokine transcription	anti-inflammatory/pro-resolving macrophage state	lung, liver
α-KG availability	TET- and KDM6A-mediated demethylation at STAT6-responsive enhancers	reparative macrophage polarization	IL-4–rich niches
oxysterols and fatty acids	LXR/PPAR-dependent co-repressor retention and p300 recruitment	homeostatic/anti-fibrotic stabilization	lung, liver
metabolic stress signals (mtROS, lactate)	persistent NF-κB/NLRP3 activity and histone lactylation	maladaptive pro-fibrotic macrophage state	IPF, CKD, post-MI

The table summarizes key metabolites and metabolic signals that couple cellular metabolism to epigenetic regulation, thereby shaping inflammatory, reparative, or maladaptive macrophage states in fibrotic disease contexts across organs.

### 2.7 Sex-dependent differences in macrophage states in homeostasis and fibrosis

Sex-related differences constitute an underappreciated but biologically relevant layer of macrophage regulation that influences immune homeostasis, inflammatory resolution and susceptibility to fibrotic disease. Across multiple organs, epidemiological and experimental studies consistently show that premenopausal females are relatively protected from progressive fibrosis, whereas this advantage is lost after menopause, implicating sex-dependent immune regulation rather than parenchymal cell–intrinsic mechanisms alone (171, 172).

Macrophages are direct targets of sex hormone signaling, as they express functional estrogen receptors ERα and ERβ as well as androgen receptor. Estrogen signaling in macrophages generally constrains NF-κB– and IRF-driven inflammatory transcription, limits inflammasome activity and promotes efferocytosis and resolution-associated programs. In contrast, androgen signaling tends to permit sustained inflammatory activation and oxidative stress responses (173, 174). These opposing transcriptional biases shape how macrophages interpret injury signals and transition between inflammatory and reparative states.

Beyond hormonal effects, females exhibit a more reactive innate immune system, characterized by enhanced PRR responsiveness and stronger cytokine induction. While this immune configuration supports effective pathogen clearance, it also increases the risk of chronic inflammation when resolution checkpoints fail (175, 176). At the macrophage level, this sex bias manifests as heightened activation thresholds in females that are normally restrained by estrogen-dependent regulatory circuits. Loss of estrogen signaling, such as after menopause, destabilizes this balance and facilitates persistence of inflammatory–remodeling macrophage states that promote fibrotic progression.

Animal models of chronic kidney, liver, lung and cardiac injury support a central role for sex-dependent macrophage regulation. Female animals or estrogen-treated males consistently display reduced macrophage accumulation, lower expression of profibrotic mediators including TGF-β, and attenuated ECM deposition compared with males. Conversely, ovariectomy or androgen supplementation exacerbates macrophage-driven inflammation and fibrosis (171, 177). Although many studies assess fibrosis at the tissue level, macrophage-specific analyses identify altered activation states and macrophage–stromal crosstalk as key mediators of these sex differences.

Human data remain limited, largely due to historical underrepresentation of sex as a biological variable in immunological studies. Nonetheless, clinical observations align with experimental findings, as postmenopausal women rapidly lose protection from fibrotic diseases and in some contexts exceed men in disease severity. This shift strongly suggests that macrophage regulatory networks become maladaptive in the absence of estrogenic control rather than through fundamental changes in macrophage identity (171, 178).

Together, these observations indicate that sex-related differences in macrophage biology arise from the integration of hormonal signaling and baseline immune organization. Estrogen-dependent restraint of inflammatory transcription supports macrophage homeostasis and resolution, whereas androgen signaling and estrogen loss favor persistent inflammatory–remodeling states that predispose tissues to fibrosis. Incorporating sex as a biological variable is therefore essential for understanding macrophage dysfunction in chronic fibrotic diseases and for the rational development of macrophage-targeted therapies.

### 2.8 Spatial organization of macrophages within normally regenerating and fibrotic tissues

Across fibrotic tissues, a recurring set of macrophage populations emerges that can now be mapped with quite high resolution in both humans and mice. Large single-cell and single-nucleus atlases show that healthy organs contain strongly imprinted resident lineages whose transcriptional profiles and niches are remarkably conserved.

In the lung, airway and alveolar territories are dominated by PPARγ^hi^ MARCO^+^ FABP4^+^ macrophages, while the interstitium is enriched in LYVE1^hi^ MRC1^hi^ FOLR2^+^ IGF1^+^ cells that sit along vessels and collagen rich stromal tracts (179). The liver hosts CD5L^+^ SPIC^+^ CLEC4F^+^ Kupffer cells with iron handling and erythrophagocytic programs, organized in periportal and midzonal niches maintained by HSCs and endothelium (180–182). In the kidney, CD81^+^ CX3CR1^+^ and LYVE1^+^ macrophages align with tubular and vascular structures and show compartment specific signatures captured by spatial and single-cell atlases (183–185). Mouse atlases identify an analogous *Timd4^+^ Lyve1^+^ Folr2^+^ Cd163^+^ Mrc1^+^ Igf1^+^* subset that recurs across heart, liver, lung, kidney and brain and corresponds well to these human resident populations, reinforcing a shared embryonic origin and niche driven maintenance program (186–188). Cross tissue immune maps such as Tabula Sapiens and HuBMAP further support this picture and define a mononuclear phagocyte continuum where tissue specific cues sculpt resident identity on a common core network (57, 189–191).

Fibrotic disease superimposes a second, monocyte-derived architecture on top of this resident scaffold. In classic mouse models of lung, liver, heart and kidney injury, CCR2-dependent Ly6C^hi^ monocytes accumulate early in damaged territories, where they upregulate genes such as *Spp1, Fn1, Mmp9, Mmp12, Lgals3, Thbs1, Chil3* and hypoxia-related modules including *Hif1a, Slc2a1* and glycolytic enzymes, while maintaining *Ccr2, Mafb* and MHC class II expression (23, 43, 49, 192–194). Over time these infiltrating cells adopt a LAM-like program that combines TREM2, SPP1, CD9, GPNMB, APOE and FABP5 and appears in fibrotic heart, liver, lung, kidney, skin and endometrium as a shared profibrotic state (34, 179, 195). Human single-cell and spatial data recapitulate this trajectory. In cirrhotic liver, TREM2^+^ CD9^+^ SPP1^+^ LGALS3^+^ scar-associated macrophages (SAMs) with FCN1^+^ MNDA^+^ monocyte heritage cluster within collagen rich septa and strongly activate HES1 and EGR2 (34, 196). In IPF and post-COVID lung disease, SPP1^hi^ MMP9^+^ CHIT1^+^ TREM2^+^ macrophages expand in fibrotic lower lobes and occupy the advancing edge of fibroblast foci (47, 50, 55, 60, 76, 197). In dilated cardiomyopathy and ischemic remodeling, FCN1^+^ CD14^+^ monocytes give rise to TREM2^+^ SPP1^+^ FABP5^+^ LGALS3^+^ macrophages in perivascular and subepicardial fibrotic regions, matching similar LAM-like states in experimental pressure overload and infarction models (198–201). Kidney atlases in CKD identify SPP1^hi^ APOE^hi^ C1QB^+^ C1QC^+^ CTSD^+^ macrophages that concentrate in the tubulointerstitium around atrophic tubules and co-localize with activated stromal cells (42, 43, 185, 202).

Spatial transcriptomics adds significant insights by showing how these transcriptional states are positioned within the fibrotic niche. Across organs, SPP1^hi^ TREM2^+^ CD9^+^ macrophages sit at the outer border of collagen scars where they are in immediate contact with fibroblasts expressing COL1A1, COL1A2, COL3A1, PDGFRA, THY1, ACTA2, POSTN and MMP14, and with endothelial and epithelial cells that supply inflammatory and growth factors (34, 50, 60, 196). Ligand receptor analyses and functional experiments point to IL-1β, PDGFα, PDGFβ, CXCL4, CSF2, WNT ligands and amphiregulin from platelets, neutrophils and fibroblasts as key drivers that consolidate the *Spp1* module and induce OPN, fibronectin, THBS1, CYR61 and semaphorin programs in these macrophages, which in turn feedback on fibroblasts to enhance proliferation, survival and matrix deposition (200, 201, 203–205). In contrast, resident LYVE1^hi^ TIMD4^+^ MARCO^+^ macrophages in lung, heart and liver tend to remain in more preserved tissue zones, maintain IGF1, CSF1, CXCL9, CXCL10 and scavenger programs and exert homeostatic or reparative functions that restrain excessive matrix accumulation, at least in experimental systems (187, 188, 206–209).

Beyond static positioning at scar borders, recent work demonstrates that macrophage transcriptional states can assemble higher-order, multilayered tissue architectures that actively organize the repair response. In skeletal muscle injury and early dystrophy, integrated single-cell and spatial transcriptomic analyses reveal that BMDM trajectories segregate into reproducible regenerative inflammation zones (RIZs) arranged concentrically around sites of myofiber damage. In this setting, inflammatory and phagocytic macrophages occupy the lesion core, including multinucleated structures associated with debris clearance, while an intermediate interface layer is enriched for macrophages expressing growth factor and resolution-associated programs marked by GPNMB and GDF15 and positioned between the necrotic focus and regenerating tissue (210, 211). Newly forming myofibers are preferentially localized adjacent to this interface zone, indicating that spatial proximity between distinct macrophage states and parenchymal cells is a defining feature of effective regeneration rather than a passive consequence of inflammation. Importantly, this architecture is coupled to a composite transcriptional regulatory logic in which a permissive reparative program linked to lipid-sensing PPARγ–RXR signaling is further refined by ATF3, identified as a candidate driver of the *Gpnmb*-associated module selectively active within the interface layer, reinforcing the notion that spatial niche and transcriptional state are mechanistically intertwined (211, 212). Disruption of this layered organization by intermittent glucocorticoid treatment results in collapse of RIZs and impaired tissue repair, underscoring that therapeutic perturbations can remodel macrophage-driven tissue architecture itself rather than simply shifting polarization markers (211).

Together, these observations support a spatially organized model in which CCR2-driven monocyte recruitment establishes a metabolically primed macrophage scaffold at sites of injury, upon which local instruction by fibroblasts, stromal cells, and platelets consolidates an OPN-centered interface state through transcriptional programs integrating PPARγ–RXR–dependent lipid sensing with context-dependent regulators such as ATF3. In parallel, niche-protected TRMs remain segregated within preserved tissue territories and retain predominantly regulatory and pro-resolving functions, emphasizing that fibrotic outcome is shaped not only by macrophage identity but by the spatial deployment of distinct macrophage programs within the tissue landscape.

## 3 Therapeutic perspectives on macrophage modulation in fibrosis

**Figure 3:**
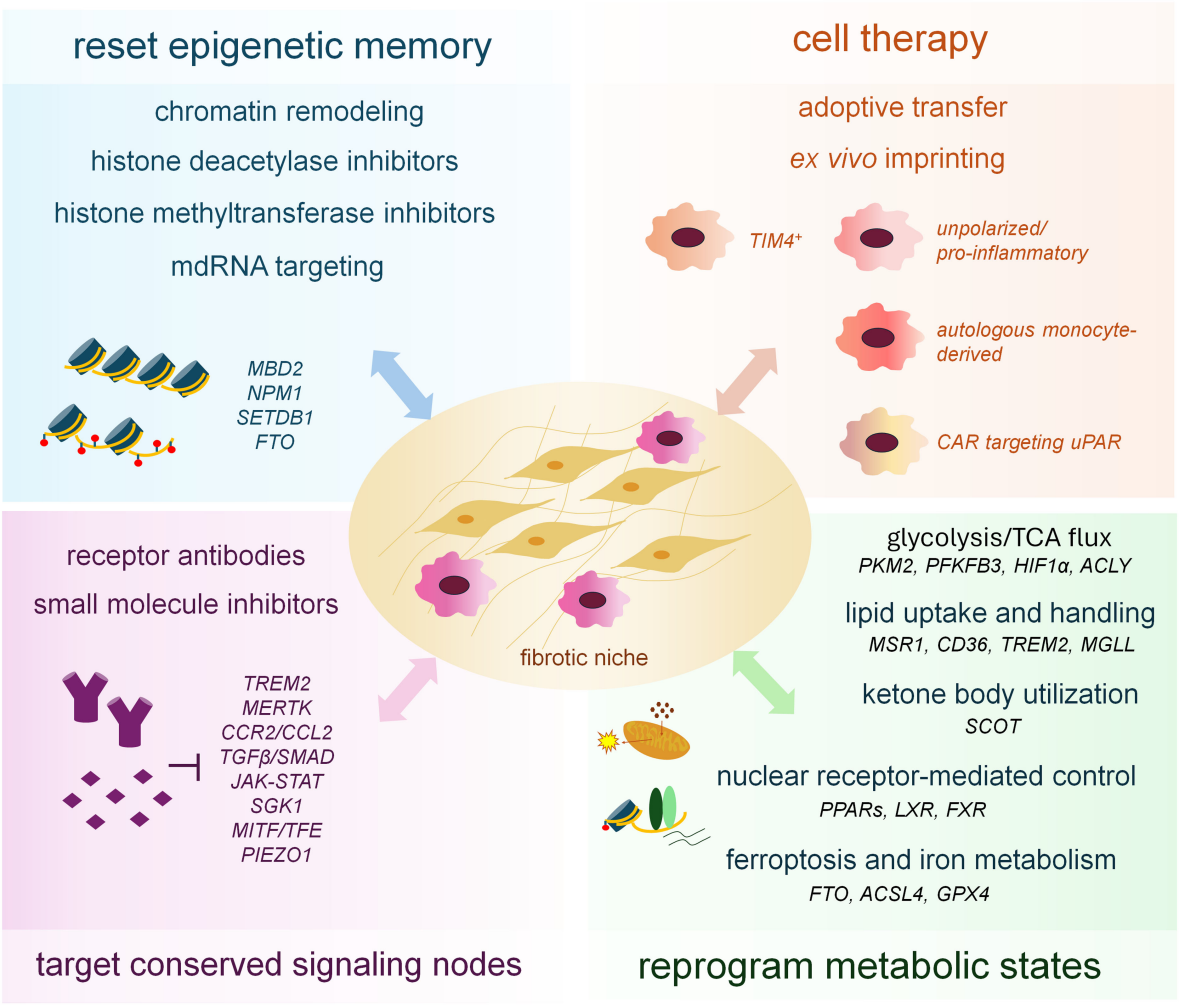
Therapeutic strategies targeting macrophage programs in fibrosis. This figure summarizes current and emerging therapeutic targets aimed at modulating macrophage function in fibrotic diseases.

Macrophages sit at the convergence of inflammatory sensing, tissue remodeling and repair in virtually all fibrotic organs. Instead of acting as a single uniform effector population, they comprise diverse subsets whose transcriptional, epigenetic and metabolic wiring determines whether they sustain chronic injury, permit scar persistence or promote resolution (30, 31). Therapeutic strategies that modulate these programs already range from small molecules targeting conserved signaling hubs, through interventions that reset epigenetic and metabolic states, to approaches that disrupt macrophage communication with stromal cells or exploit engineered macrophages as cell therapies. Recent studies in lung, liver, kidney, skin and heart illustrate how these concepts are beginning to translate into mechanistically informed interventions, and they define the main directions in macrophage-centered antifibrotic therapy (**Figure 3**).

### 3.1 Targeting conserved transcriptional and signaling nodes in macrophage-mediated fibrosis

Single-cell studies in human lung, liver and kidney fibrosis consistently identify macrophage populations that express TREM2, OPN, LGALS3, GPNMB and a panel of lipid-handling genes, and that localize along fibrotic septa or fibroblastic foci (30, 213, 214). These populations integrate persistent cytokine stimulation, metabolic stress and altered matrix mechanics into relatively stable profibrotic identities. Because they sit at the intersections where TGF-β, JAK–STAT, AP-1, MITF/TFE and nuclear receptor pathways converge, they offer several therapeutic entry points.

TGF-β–SMAD2/3 remains the best-characterized upstream driver of both HSC activation and profibrotic macrophage programming in liver disease (215, 216). In experimental models, TGF-β receptor kinase inhibition or SMAD3 deficiency reduces collagen deposition and lowers expression of *Spp1* and *Lgals3* in liver macrophages that reside in scarred regions (203, 217). Similar macrophage-directed effects have been described in the lung, where TGF-β-rich environments drive OPN-producing and MMP-expressing macrophage states that amplify fibroblast activation in IPF, bleomycin-induced, and radiation-induced lung fibrosis (46, 218–221). These observations support the idea that TGF-β targeting does not only disarm myofibroblasts but also destabilizes profibrotic macrophage states.

These observations support the concept that therapeutic TGF-β blockade may disrupt profibrotic macrophage states in patients as well. Clinical studies of TGF-β–targeting agents, including prolonged-release pirfenidone, have shown fibrosis regression and improved hepatic function in patients with alcoholic liver fibrosis (PROMETEO trial) (222), while pharmacokinetic data in cirrhotic subjects demonstrated acceptable exposure and tolerability (223). Moreover, a phase II trial of hydronidone in chronic hepatitis B reported significant histological fibrosis improvement with good safety (224), and montelukast reduced liver stiffness and fibrosis biomarkers (TNF-α, TGF-β1, hyaluronic acid) in MASH patients (225).

AP-1 factors are key organizers of profibrotic transcription. In Fra-2–driven lung fibrosis, macrophage-derived collagen VI is a major effector of their fibroblast-activating capacity. Inhibiting Fra-2 lowers collagen VI in these cells, reduces their stimulatory output and alleviates fibrosis in both genetic and bleomycin models, consistent with Fra-2 and collagen VI co-localization in IPF macrophages (226). This illustrates how a single transcription factor can reprogram the macrophage–matrix interface and opens an AP-1 dependent axis for drug development.

A growing number of studies highlight macrophage-specific receptors as particularly attractive targets because they offer a way to interfere with pathogenic macrophage states while sparing other cell types. TREM2 is highly expressed on monocyte-derived alveolar macrophages in fibrotic mouse lungs and in lung macrophages from patients with IPF. Deletion or knockdown of TREM2 disrupts intracellular survival signaling, promotes apoptosis of these macrophages and alleviates lung fibrosis in several models, while a lipid ligand present in the alveolar milieu appears to sustain their survival and profibrotic activity (227). A similar principle emerges in metabolic liver disease, where MerTK signaling in macrophages promotes an ERK–TGF-β1 pathway that activates HSCs and accelerates fibrosis in MASH models. Myeloid-specific MERTK targeting reduces fibrosis and provides a mechanistic explanation for human genetic data linking hypomorphic MERTK variants to protection from MASH fibrosis (228).

Cytokine-driven pathways that converge on STAT3 and related transcription factors also shape macrophage behavior in fibrotic settings. IL-6, IL-11 and OSM are enriched at macrophage–fibroblast interfaces in experimental and human disease (34, 229, 230). Inhibition of these pathways reduces profibrotic gene expression in both macrophages and HSCs in liver organoid systems and diminishes experimental fibrosis in the liver (231, 232). SGK1 has recently been identified as a key kinase shaping macrophage behavior in lung fibrosis. In bleomycin- and LPS-injury models, SGK1^hi^ macrophages adopt a reparative, growth-factor enriched profile and secrete CCL9 and TGF-β, which together promote Th17 influx and fibroblast activation (233). Pharmacologic SGK1 inhibition potentially redirects macrophage programming toward a less fibrogenic state, remodels the inflammatory environment and significantly reduces fibrosis (234).

Mechanosensitive pathways connect matrix stiffness to macrophage transcriptional state. In fibrotic skin and other mechanically stressed wounds, a subset of myeloid cells switches between SEPP1^+^MRC1^+^EGR1^+^ regenerative profiles in compliant tissue and Thrombospondin^+^CD86^+^ profibrotic profiles in stiff microenvironments. Interfering with mechanical signaling in these macrophages shifts transcriptional programs toward regenerative, pro-healing states and leads to thinner, less aligned collagen bundles that resemble unwounded skin architecture (235).

In the kidney, PIEZO1 in myeloid cells is required for full development of renal fibrosis. *Piezo1* deletion reduces macrophage accumulation and pro-inflammatory activation and protects against fibrosis after UUO or folic acid injury, in part through effects on CCL2–CCR2 and Notch signaling (236). These examples show how conserved transcriptional and signaling nodes in macrophages can be targeted not only through classic cytokine and receptor pathways but also through mechanosensitive machinery that is highly relevant in stiff fibrotic tissues.

### 3.2 Epigenetic reprogramming as a strategy to reset macrophage state

The stability of profibrotic macrophage states reflects persistent cytokine and metabolic signaling, but also the establishment of a durable enhancer architecture that reinforces expression of genes such as OPN, TREM2, GPNMB and LGALS3. SMAD3, STAT3 and AP-1 cooperate with p300/CBP and SWI/SNF complexes to deposit activating histone marks at super-enhancers that define these states, while removal of repressive marks completes the transition. Because these chromatin structures act as the molecular substrate that locks BMDMs onto fibrogenic trajectories, epigenetic interventions are attractive candidates for resetting macrophage identity, especially in chronic disease where transcription factor targeting alone may have limited durability.

Several recent studies exemplify how epigenetic regulators in macrophages control organ fibrosis. In pulmonary fibrosis, MBD2 expression is elevated in macrophages from patients and bleomycin-treated mice. MBD2 binds and represses the *Ship* promoter, which amplifies PI3K–Akt signaling and drives a shift toward a TGF-β-rich, pro-fibrotic activation profile. Macrophage-specific MBD2 deficiency lowers TGF-β output, reduces the accumulation of these profibrotic macrophage states and protects mice from lung injury and scarring, and intratracheal delivery of *Mbd2*-targeting siRNA recapitulates this benefit (237). This work directly links a specific methyl-CpG binding protein in macrophages to fibrotic outcome and illustrates how targeted epigenetic modulation can modify macrophage behavior *in vivo*.

The heart provides a complementary example in which an epigenetic factor integrates metabolism and inflammation. After myocardial infarction, NPM1 expression increases in peripheral blood mononuclear cells from patients and correlates with adverse outcomes. In mouse models, macrophage-specific deletion of *Npm1* reduces infarct size, improves angiogenesis and limits adverse remodeling. Mechanistically, oligomeric NPM1 recruits the histone demethylase KDM5b to the *Tsc1* promoter, reduces H3K4me3 and suppresses TSC1 expression, which favors mTOR-dependent glycolysis and an inflammatory profile. Loss or inhibition of NPM1 shifts macrophage metabolism toward OXPHOS, enhances reparative functions and reduces fibrosis (238). These findings emphasize that epigenetic factors can be used to redirect macrophage metabolism and phenotype simultaneously.

Histone methylation in macrophages has emerged as another important determinant of fibrotic outcome. In a model of chronic rejection and tumor-associated fibrosis, macrophage SETDB1 drives a profibrogenic FN1^+^ macrophage state. FN1 induced through a CCR2–CREB–SETDB1 axis promotes profibrotic gene expression in both fibroblasts and macrophages via integrin and paired immunoglobulin receptor A signaling. Myeloid-specific deletion of *Setdb1* inhibits fibrosis in allografts and tumor tissues, identifying histone methylation in macrophages as a potential therapeutic target across several fibrotic contexts (239).

Epigenetic mechanisms also converge with iron and lipid metabolism in kidney fibrosis. In CKD, genetic or pharmacologic inhibition of the RNA demethylase FTO reduces collagen deposition, lipid peroxidation and ferroptosis-related markers after UUO. FTO blockade downregulates ACSL4, a key ferroptosis driver, and directly suppresses TGFBI. These changes are accompanied by a decline in profibrotic macrophage populations within the kidney and an overall reduction in scarring, indicating that RNA methylation status can influence macrophage recruitment and activation indirectly through metabolic and stromal pathways (240). In parallel, upregulation of the Src family kinase HCK in pro-inflammatory kidney macrophages interferes with autophagy by interacting with ATG2A and CBL. HCK deletion or pharmacological inhibition restores autophagy flux, reduces pro-inflammatory polarization and attenuates renal inflammation and fibrosis in several models (241). Although not classical chromatin modifiers, these signaling molecules influence transcriptional landscapes through effects on autophagy, protein stability and downstream gene expression.

Work in liver disease complements these findings by showing how macrophage epigenetics and autophagy jointly regulate fibrotic outcomes. Macrophage autophagy protects against liver fibrosis, in part through LC3-associated phagocytosis that limits inflammatory signaling (149, 242). In cirrhosis, macrophage-targeted therapies that enhance autophagy reduce fibrogenic cytokine production and alter cytokine patterns in patients who receive autologous macrophage infusions (243). Collectively, these examples show that macrophage epigenetic and related homeostatic processes are not static features, but dynamic levers that can be pharmacologically manipulated to reset macrophage state and, by extension, the fibrotic trajectory of the tissue.

### 3.3 Metabolic reprogramming to redirect macrophage fate

Metabolic state has a powerful influence on macrophage phenotype, and in fibrosis it functions as both a driver and a stabilizer of profibrotic programs. Classical immunometabolism studies showed that inflammatory macrophages depend on enhanced glycolysis, altered TCA flux and NO production, whereas anti-inflammatory macrophages favor FAO and OXPHOS (244). In chronic liver disease, these principles take on a specific shape. Lipid-laden Kupffer cells and infiltrating monocyte-derived macrophages accumulate diacylglycerol, ceramide and oxidized lipoproteins through scavenger receptors such as MSR1 and CD36, and this accumulation promotes a pro-inflammatory and profibrogenic phenotype (245, 246). Mice lacking *Msr1* are less prone to hepatic inflammation and fibrosis, underscoring the link between lipid uptake, macrophage activation and fibrogenesis (245).

Targeting specific metabolic enzymes in macrophages already produces antifibrotic effects in preclinical models. Inhibition of monoacylglycerol lipase in myeloid cells shifts lipid metabolism away from arachidonic acid and pro-inflammatory prostaglandins toward an anti-inflammatory 2-arachidonoylglycerol profile. This shift reduces Ly6C^hi^ macrophages and increases Ly6C^lo^ restorative macrophages, protects from cholestatic injury and biliary fibrosis and promotes regression of established fibrosis (247, 248).

The hepatocyte–macrophage acetoacetate shuttle relies on ketone body production by hepatocytes and their oxidation in macrophages through SCOT. Acetoacetate use in macrophages dampens fibrogenic signaling, and exogenous acetoacetate reduces pathological matrix deposition in diet-induced liver injury. When SCOT is selectively deleted in macrophages, glycosaminoglycan metabolism becomes dysregulated and fibrosis accelerates, highlighting ketone handling as a determinant of macrophage behavior in the injured liver (249). This concept reinforces that not only intrinsic macrophage metabolism, but also intercellular metabolite shuttles influence fibrotic outcome.

At the other end of the phenotypic spectrum, ferroptosis and lipid peroxidation can drive inflammation and fibrogenesis. In radiation-induced lung fibrosis, ferroptosis inhibition reduces TGF-β1 levels and attenuates fibrotic remodeling (250). In kidney obstruction, inhibition of FTO decreases ferroptosis markers and lipid peroxidation and is accompanied by a decline in profibrotic macrophage populations within the obstructed kidney (240). Macrophages respond to iron and lipid peroxidation with changes in polarization status, and some of these responses can be harnessed therapeutically, as illustrated by the association between iron-altered macrophage states and steatohepatitis and fibrosis in the liver (251, 252).

Nuclear receptor signaling links these metabolic features to transcriptional control. LXR and FXR pathways regulate cholesterol handling in HSCs, sinusoidal endothelial cells and macrophages. Activation of LXR or FXR in preclinical models reduces inflammation and fibrosis and can modulate macrophage phenotypes, although cell-type specificity is important (59, 253–257). PPAR agonists influence both hepatocyte lipid metabolism and macrophage activation. PPARα activation improves endothelial dysfunction and reduces portal hypertension and fibrosis in cirrhotic rats (258), and thiazolidinediones such as pioglitazone inhibit collagen synthesis and HSC activation and show beneficial effects in MASH patients (259, 260). Selective and pan-PPAR agonists have differential effects on experimental steatohepatitis and hepatic macrophages, which is now being systematically explored in trials such as NATIVE, DESTINY and others (261–264).

Taking together, these data show that macrophage fate in fibrosis is tightly linked to local metabolic pressures. Lipid overload, altered ketone body flux, iron imbalance, ferroptosis and nutrient-sensing pathways shape the transcriptional architecture of macrophage populations in ways that either support or constrain fibrogenesis. Therapeutic strategies that correct these metabolic derangements or selectively redirect macrophage metabolism toward oxidative, pro-resolving states have the potential to synergies with transcriptional and epigenetic interventions and to increase the likelihood of durable fibrosis regression.

### 3.4 Interrupting macrophage–stromal cell communication

Fibrosis persists not simply because macrophages acquire profibrotic programs, but because these programs become embedded in self-reinforcing circuits that link macrophages to fibroblasts, endothelial and epithelial cells and the ECM. Interrupting these communication loops offers a way to weaken macrophage pathogenicity without requiring complete elimination of these cells.

In the lung, chemokine networks have been exploited to intercept macrophage recruitment and activation. A new mouse model that combines bleomycin and thoracic irradiation produces continuous alveolitis-mediated fibrosis that closely mimics clinical IPF. ScRNA-seq and functional experiments show that the CCL2–CCR2 axis drives polarization of inflammatory macrophages that foster alveolitis and myofibroblast activation. Nanostructured tetrahedral nucleic acids carrying siRNA against *Ccr2* selectively target these macrophages, reduce their accumulation, prevent myofibroblast activation and lead to disease remission (265). Similar experiments in the liver resulted in attenuation of cirrhosis (266).

P2Y12 is another macrophage receptor that links TGF-β signaling to a macrophage-to-myofibroblast transition in CKD. P2Y12 expression in macrophages is induced by TGF-β and promotes expression of α-smooth muscle actin through SMAD3. Genetic silencing or pharmacological inhibition of P2Y12 blocks this transition and reduces fibrosis in patients and in UUO models (267).

In SSc, mesenchymal stem cell (MSC) therapy provides another example of interrupting macrophage–stromal circuits. IFN-γ and TNF-α pretreated MSCs alleviate bleomycin-induced skin fibrosis. Macrophages are the main profibrotic immune cells in this model, and MSCs reduce the accumulation of maturing CCR2^hi^ macrophages by limiting CCL2 production from fibroblasts and macrophages (268). This shows how tweaking the fibroblast output of chemokines can indirectly reprogram macrophage composition in fibrotic skin.

Liver studies continue this theme in a different direction. CYR61 produced by hepatocytes polarizes infiltrating monocytes toward a pro-inflammatory and profibrotic phenotype through IRAK4–SYK–NF-κB signaling and induces PDGFα and PDGFβ expression in macrophages. Antibody blockade of CYR61 reduces liver inflammation and fibrosis in MASH models and weakens the macrophage–HSC axis that sustains scarring (269). Across these examples, the common theme is that macrophages and stromal cells constantly exchange signals that reinforce fibrosis, and that carefully chosen targets can dismantle these loops without completely silencing innate immunity.

### 3.5 Cell-based therapeutic strategies to reprogram macrophages in fibrosis

Alongside pharmacological approaches, cell-based strategies offer a way to directly replace or reprogram dysfunctional macrophage populations in fibrotic organs. The liver has progressed furthest toward clinical translation. Monocyte-derived macrophages have been developed as a Good Manufacturing Practice compatible product, and it has been shown that monocytes from patients with cirrhosis can be differentiated *ex vivo* into macrophages with a phenotype comparable to those from healthy donors (243). A first-in-human phase 1 study demonstrated the safety of autologous macrophage infusion and suggested improvement in non-invasive fibrosis markers in some participants. The recent MATCH01 phase 2 trial in compensated cirrhosis again confirmed safety and hinted at fewer liver-related serious adverse events and deaths in the macrophage-treated group, although the primary MELD endpoint was not met and the cohort was relatively small (243). Together, these trials show that macrophage therapy is feasible and well tolerated in patients with advanced liver disease, even though robust efficacy still needs to be established.

Preclinical work explores how macrophages can be educated before transfer. In experimental liver fibrosis, unpolarized and pro-inflammatory BMDMs reduce fibrosis when infused during the resolution phase. These macrophages decrease HSC numbers and activation, promote HSC apoptosis and recruit Ly6C^low^ restorative macrophages that secrete MMPs and HGF (36). In CKD, adoptive transfer of iron-replete macrophages that have a restored labile iron pool decreases oxidative stress, lowers pro-inflammatory cytokines and attenuates fibrosis (270). In MASH, infusion of TIM4^+^ macrophages improves efferocytosis, enhances IL-10–dependent inactivation of HSCs and slows the transition from steatosis to fibrosis (271).

Engineered macrophages extend this concept. CAR-macrophages that express a chimeric antigen receptor targeting uPAR on HSCs have shown robust antifibrotic efficacy in mouse models. Adoptive transfer of these CAR-macrophages reduces collagen deposition, improves liver function and reshapes the hepatic immune microenvironment, combining direct clearance of uPAR^+^ HSCs with induction of antifibrotic T cell responses (272). In experimental autoimmune encephalomyelitis, macrophages pre-programmed with IGF2 adopt an OXPHOS-committed, anti-inflammatory phenotype with high PD-L1 expression, increase regulatory T cells after transfer and ameliorate disease (273). Although this last study sits outside classical organ fibrosis, it illustrates how *ex vivo* imprinting of metabolism and signaling can preset macrophage responsiveness *in vivo*.

Altogether, macrophage-centered cell therapies are moving from proof-of-concept toward more refined, mechanism-based designs. Engineered or pre-educated macrophages can be tailored to promote efferocytosis, enhance matrix degradation, restrain HSC activation or even directly eliminate pathogenic stromal cells. Challenges remain, including long-term engraftment, maintenance of a reparative phenotype in hostile fibrotic niches and organ-specific homing, but the combination of early clinical data and rapidly evolving preclinical work suggests that macrophage-directed cell therapies will become an important complement to pharmacological strategies aimed at transcriptional, epigenetic and metabolic points in fibrosis.

## 4 Conclusions

**Figure 4:**
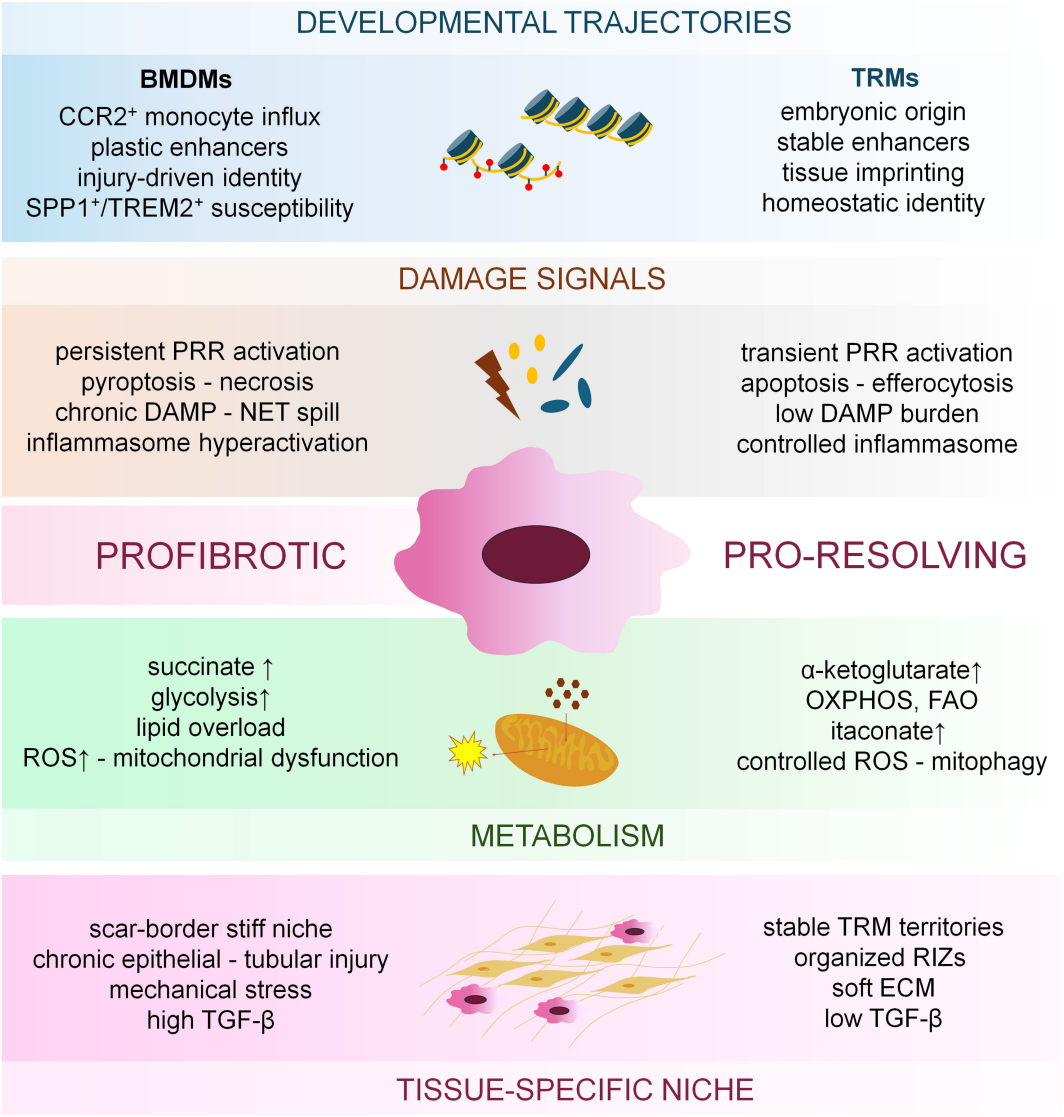
Multilayered regulatory inputs guiding macrophage fate toward resolution or fibrosis. The figure summarizes four regulatory layers that collectively shape macrophage behavior during tissue injury. (1) Ontogeny and developmental encoding (blue): Embryo-derived TRMs maintain stable enhancer landscapes and niche-imprinted homeostatic programs, whereas BMDMs display more plastic chromatin and greater susceptibility to pro-fibrotic reprogramming. (2) Damage-associated signals (brown): Transient, low-intensity inflammatory cues favor resolution, while persistent DAMPs, NETs and inflammasome activation promote chronic activation and fibrotic macrophage states. Metabolic inputs (green): Oxidative and lipid-based metabolism supports reparative phenotypes, whereas glycolytic bias, mitochondrial stress and disrupted lipid handling reinforce fibrotic pathways. (4) Tissue-specific niche cues (pink): Intact epithelial, endothelial and stromal signals maintain regulatory macrophage functions, whereas chronic injury and increased matrix stiffness create microenvironments that drive macrophages toward pro-fibrotic activation.

Macrophages rely on a multilayered gene-regulatory system in which LDTFs, signal-dependent transcriptional pathways, chromatin accessibility, metabolic state, and microenvironmental inputs interact to produce a broad spectrum of activation states. This multidimensional architecture is the basis of macrophage plasticity. It enables rapid adaptation to inflammatory cues and supports the transition toward resolution and tissue repair. At the same time, it also creates vulnerability. When inflammatory signals persist, when stromal feedback becomes dysregulated, or when metabolic or mechanical conditions shift toward pathological extremes, these regulatory layers can align in ways that stabilize maladaptive transcriptional states. Fibrosis, in this sense, represents a failure of regulatory flexibility where macrophages become locked into pro-fibrotic phenotypes by sustained enhancer activation, disrupted resolution programs, and tissue-specific constraints (**Figure 4**).

Although the core regulatory modules underlying these transitions are conserved, the outcomes are strongly shaped by the metabolic, structural, and immunological features of each organ. Hypoxia, lipid overload, epithelial injury, complement activation, stromal feedback, and mechanical forces each impose specific transcription factor interplay, chromatin remodeling, and macrophage metabolism. As a result, macrophage heterogeneity in fibrosis reflects tissue-conditioned adaptations of a shared regulatory scaffold, giving rise to similar pathological endpoints through distinct regulatory trajectories.

While this review has focused on transcriptional and chromatin-based mechanisms as the primary drivers of macrophage behavior, it is important to acknowledge that additional post-transcriptional and post-translational regulatory layers further complicate this landscape. Mechanisms such as RBP-mediated control of mRNA stability, alternative splicing, microRNA-driven repression or amplification of signaling pathways, stimulus-dependent translation, and dynamic protein modifications including phosphorylation, ubiquitination, acetylation, and selective proteolysis can fine-tune the magnitude, timing, and coherence of macrophage responses independently of enhancer architecture. These layers do not redefine macrophage identity, but they modulate how transcriptional programs are executed in real time, adding further complexity to the already intricate gene-regulatory networks governing macrophage plasticity in fibrosis.

Despite rapid progress made possible by single-cell and spatial multi-omics, critical knowledge gaps persist. Much of our mechanistic insight derives from mouse models, which often employ acute or simplified injury systems that differ substantially from the chronic, multifactorial etiologies characteristic of human disease. Early phases of fibrotic remodeling remain particularly inaccessible in patients, making it difficult to determine which regulatory events are causal and which reflect downstream adaptation. Moreover, aging introduces additional complexity: age-associated epigenetic drift, metabolic inflexibility, impaired efferocytosis, and low-grade inflammation alter macrophage behavior and reshape tissue niches, yet these processes remain poorly integrated into current models of macrophage dysfunction.

Addressing these gaps will require high-resolution temporal studies in human tissues, perturbation-based approaches to test regulatory dependencies in primary human macrophages and harmonized cross-species analyses to distinguish conserved pathways from model-specific adaptations. From a translational perspective, recognizing fibrosis as the product of gene-regulatory networks reshaped by tissue-specific cues suggests that effective therapies will need to intervene at the level of these regulatory intersections. Targeted modulation of SMAD and STAT3 signaling, reprogramming of macrophage metabolism, selective epigenetic interventions, and disruption of macrophage–stromal feedback loops represent promising strategies. However, therapeutic precision will depend on accounting for organ-specific microenvironments, metabolic constraints, spatial localization, and age-related alterations in macrophage responsiveness.

Continued progress in single-cell and spatial technologies will be essential for defining which regulatory features of fibrotic macrophages are causal, conserved, and therapeutically relevant. A more integrated understanding of how developmental origin, microenvironmental signals, chromatin state, metabolism, and aging collectively shape macrophage function is likely to improve the identification of realistic intervention points and guide the development of more effective antifibrotic strategies.

## 5 Literature selection strategy

This review is based on a targeted, narrative literature search performed in PubMed and Google Scholar. We primarily focused on studies published between 2015 and 2025, while including earlier seminal work where necessary to establish conceptual frameworks. Search terms combined macrophage-related keywords with fibrosis- and tissue-specific terms (e.g. macrophage, fibrosis, gene regulation, epigenetics, metabolism, lung, liver, kidney, heart), as well as technology-oriented keywords (single-cell RNA-seq, spatial transcriptomics, multi-omics). Studies were selected based on mechanistic relevance to macrophage transcriptional regulation and tissue context, with particular emphasis on recent human and integrative single-cell and spatial datasets rather than formal systematic inclusion criteria.

## Glossary

ACLY: ATP-Citrate Lyase
ACOD1: Aconitate Decarboxylase 1
AKI: Acute Kidney Injury
AP-1: Activator Protein 1
ATF: Activating Transcription Factor
BMDM: Bone Marrow-Derived Macrophage
BRG: Brahma-Related Gene
C/EBP: CCAAT-Enhancer-Binding Protein
CAR: Chimeric Antigen Receptor
CAR-M: Chimeric Antigen Receptor–Engineered Macrophage
CBP: CREB-Binding Protein
CCL2: C-C Motif Chemokine Ligand 2
CCR2: C-C Motif Chemokine Receptor 2
CKD: Chronic Kidney Disease
CLR: C-type Lectin Receptor
COPD: Chronic Obstructive Pulmonary Disease
CSF1: Colony-Stimulating Factor 1
CSF1R: Colony-Stimulating Factor 1 Receptor
DAMP: Damage-Associated Molecular Pattern
EAE: Experimental Autoimmune Encephalomyelitis
ECM: Extracellular Matrix
EMT: Epithelial–Mesenchymal Transition
ER: Endoplasmic Reticulum
ERK: Extracellular Signal-Regulated Kinase
FAO: Fatty Acid Oxidation
FN1: Fibronectin 1
FTO: Fat Mass and Obesity-Associated Protein
FXR: Farnesoid X Receptor
GDF: Growth Differentiation Factor
GM-CSF: Granulocyte-Macrophage Colony-Stimulating Factor
GMP: Good Manufacturing Practice
GSK3β: Glycogen Synthase Kinase 3 Beta
HCK: Hematopoietic Cell Kinase
HDAC: Histone Deacetylase
HES1: Hairy and Enhancer of Split 1
HGF: Hepatocyte Growth Factor
HIF: Hypoxia-Inducible Factor
HMGB: High Mobility Group Box
HSC: Hepatic Stellate Cell
IFN: Interferon
IGF: Insulin-like Growth Factor
IL: Interleukin
IPF: Idiopathic Pulmonary Fibrosis
IRAK4: Interleukin-1 Receptor-Associated Kinase 4
IRF: Interferon Regulatory Factor
JAK: Janus Kinase
KDM: Lysine-Specific Demethylase
KMT2D: Lysine Methyltransferase 2D
LAM: Lipid-Associated Macrophage
LC3: Microtubule-Associated Protein 1A/1B Light Chain 3B
LDTF: Lineage-Determining Transcription Factor
LPS: Lipopolysaccharide
LXR: Liver X Receptor
MAF: v-maf MusculoAponeurotic Fibrosarcoma Oncogene Homolog
MAFB: v-maf MusculoAponeurotic Fibrosarcoma Oncogene Homolog B
MAPK: Mitogen-Activated Protein Kinase
MASH: Metabolic dysfunction–Associated SteatoHepatitis
MASLD: Metabolic Dysfunction–Associated Steatotic Liver Disease
MBD2: Methyl-CpG–Binding Domain Protein 2
MELD: Model for End-stage Liver Disease
MITF/TFE: Microphthalmia family of Transcription Factors/Transcription Factor E family
MMP: Matrix Metalloproteinase
MSC: Mesenchymal Stem Cell
mTORC1: Mechanistic Target of Rapamycin Complex 1
NET: Neutrophil Extracellular Trap
NF-κB: Nuclear Factor κB
NFAT5: Nuclear Factor of Activated T Cells 5
NLR: NOD-Like Receptor
NLRP3: NOD-, LRR- and Pyrin Domain–Containing Protein 3
NLRX1: NOD-Like Receptor X1
NO: Nitric Oxide
NPM1: Nucleophosmin 1
NR: Nuclear Receptor
OPN: Osteopontin
OXPHOS: OXidative PHOSphorylation
PD-L1: Programmed Death-Ligand 1
PDGF: Platelet-Derived Growth Factor
PDGFR: Platelet-Derived Growth Factor Receptor
PERK: PKR-like Endoplasmic Reticulum Kinase
PFKFB3: 6-Phosphofructo-2-Kinase/Fructose-2,6-Bisphosphatase 3
PI3K: Phosphoinositide 3-Kinase
PKM2: Pyruvate Kinase M2
PPAR: Peroxisome Proliferator-Activated Receptor
PPP: Pentose Phosphate Pathway
PRR: Pattern-Recognition Receptor
PSC: Primary Sclerosing Cholangitis
PU.1: Purine Rich Box 1
RAGE: Receptor for Advanced Glycation End-products
RIZ: Regenerative Inflammation Zone
ROS: Reactive Oxygen Species
RUNX: Runt-Related Transcription Factor
SAM: Scar-Associated Macrophage
SCOT: Succinyl-CoA:3-Oxoacid CoA Transferase
scRNA-seq: Single-Cell RNA Sequencing
SETDB1: SET Domain Bifurcated 1
SGK1: Serum/Glucocorticoid-Regulated Kinase 1
SHIP: SH2-domain–Containing Inositol Phosphatase 1
siRNA: Small Interfering RNA
SMAD: Sma and Mad homolog
SSc: Systemic Sclerosis
STAT: Signal Transducer and Activator of Transcription
SWI/SNF: SWItch/Sucrose Non-Fermentable
TCA: Tricarboxylic Acid Cycle
TET: Ten-Eleven Translocation
TG2: Transglutaminase 2
TGF-β: Transforming Growth Factor β
TGFBI: Transforming Growth Factor Beta-Induced
TLR: Toll-Like Receptor
TMAO: Trimethylamine-N-oxide
TNF: Tumor Necrosis Factor
TRIF: TIR-domain–containing Adaptor-Inducing Interferon-β
TRM: Tissue-Resident Macrophage
uPAR: Urokinase-Type Plasminogen Activator Receptor
UUO: Unilateral Ureteral Obstruction
XBP1: X-box Binding Protein 1
α-KG: α-ketoglutarate.
